# High-Performance Top-Gate Thin-Film Transistor with an Ultra-Thin Channel Layer

**DOI:** 10.3390/nano10112145

**Published:** 2020-10-28

**Authors:** Te Jui Yen, Albert Chin, Vladimir Gritsenko

**Affiliations:** 1 Department of Electronics Engineering, National Chiao Tung University, Hsinchu 300, Taiwan; yenrick42269.ee05g@g2.nctu.edu.tw; 2 Rzhanov Institute of Semiconductor Physics, Siberian Branch, Russian Academy of Sciences, 630090 Novosibirsk, Russia; grits@isp.nsc.ru; 3 Novosibirsk State University, 630090 Novosobirsk, Russia; 4 Novosibirsk State Technical University, 630020 Novosibirsk, Russia

**Keywords:** thin-film transistor, SnO_2_, TFT, integrated circuit, monolithic, 3D IC, brain-mimic

## Abstract

Metal-oxide thin-film transistors (TFTs) have been implanted for a display panel, but further mobility improvement is required for future applications. In this study, excellent performance was observed for top-gate coplanar binary SnO_2_ TFTs, with a high field-effect mobility (*μ_FE_*) of 136 cm^2^/Vs, a large on-current/off-current (I_ON_/I_OFF_) of 1.5 × 10^8^, and steep subthreshold slopes of 108 mV/dec. Here, *μ_FE_* represents the maximum among the top-gate TFTs made on an amorphous SiO_2_ substrate, with a maximum process temperature of ≤ 400 °C. In contrast to a bottom-gate device, a top-gate device is the standard structure for monolithic integrated circuits (ICs). Such a superb device integrity was achieved by using an ultra-thin SnO_2_ channel layer of 4.5 nm and an HfO_2_ gate dielectric with a 3 nm SiO_2_ interfacial layer between the SnO_2_ and HfO_2_. The inserted SiO_2_ layer is crucial for decreasing the charged defect scattering in the HfO_2_ and HfO_2_/SnO_2_ interfaces to increase the mobility. Such high *μ_FE_*, large I_ON_, and low I_OFF_ top-gate SnO_2_ devices with a coplanar structure are important for display, dynamic random-access memory, and monolithic three-dimensional ICs.

## 1. Introduction

The development of high-performance transistors has been continuously pursued for more than seven decades, since the transistor was invented in 1947. The metal-oxide thin-film transistor (TFT) was invented in 1964 [1], and had the important merits of low-temperature fabrication, a simple process for mass production, and visible light transparency [1,2,3,4,5,6,7,8,9,10,11,12,13,14,15,16,17,18,19,20,21,22,23,24,25,26]. Moreover, metal-oxide TFTs have widely diverse applications, such as in active matrix organic light emitting diodes [2,3], flexible electronics [4,5,6,7], and gas sensors [8,9,10]. By applying a high-mobility channel material and high-dielectric-constant (high-κ) gate dielectric, metal-oxide TFT can also be used in high-speed low-power monolithic three-dimensional (3D) integrated circuits (ICs) [11,12,13,14,15,16]. Furthermore, the wide energy bandgap, excellent field-effect mobility (*μ_FE_*) at high temperatures, and low leakage current of metal-oxide TFTs are especially important for high-temperature electronics [17] and dynamic random-access memory (DRAM) access transistors. In this paper, we report a top-gate SnO_2_ TFT that uses a combined HfO_2_ and SiO_2_ stack as a gate dielectric layer and an SnO_2_ channel layer. The top-gate TFT structure is more favorable than the bottom-gate device, owing to its high performance and easy integration in forming an IC. This top-gate device, with an SiO_2_ interfacial layer between HfO_2_ and SnO_2_, exhibited an excellent device performance, with a remarkably high *μ_FE_* of 136 cm^2^/Vs, a large on-/off-current (I_ON_/I_OFF_) of 1.5 × 10^8^, a sharp subthreshold slope (SS) of 108 mV/dec, and a much better resistance to moisture than the bottom-gate SnO_2_ TFT. Here, the SiO_2_ interfacial layer with a thickness of 3 nm is the key factor in decreasing the charged defect scattering inside HfO_2_ and increasing the *μ_FE_*. Such high-performance TFTs are crucial for future-generation high-resolution displays, DRAM access transistors, high interconnect-density monolithic 3D ICs, and 3D brain-mimicking ICs [11,12,13], where the down-scaling of silicon ICs is expected to be ended at an equivalent node around 1 nm within ten years.

## 2. Materials and Methods

P-type silicon wafers with ~10 ohmic-cm resistivity were used as substrates. A standard IC cleaning process was applied to remove the particles and native oxide from the silicon substrate. Then, an SiO_2_ layer with a thickness of 300 nm was formed on the substrate, and was used as an inter-metal-dielectric layer of the IC. Thereafter, a 4.5 nm SnO_2_ layer was deposited through reactive sputtering with a Sn target under a pressure of 7.6 × 10^−3^ torr, a mixture of O_2_/Ar gas flow at 20/24 sccm, and a DC power of 50 W. The deposited SnO_2_ layer was subjected to post-annealing at 350 °C in ambient air for 30 min. Next, 30 nm low work function aluminum Schottky source and drain electrodes [27,28] were deposited and patterned. Subsequently, a 3 nm SiO_2_ layer and a 50 nm high-κ HfO_2_ gate dielectric were deposited on the SnO_2_ layer through physical vapor deposition. Finally, a 30 nm Ni top-gate electrode was created using electron-beam evaporation and patterning. The gate length and width are 50 and 400 μm, respectively. Material analyses through X-ray photoelectron spectroscopy (XPS), secondary ion mass spectrometry (SIMS), and high-resolution transmission electron microscopy (TEM) were performed using Thermo Nexsa (Thermo Fisher Scientific Inc., Waltham, MA, USA), CAMECA IMS-6fE7 (CAMECA, Gennevilliers, France), and FEI Talos F200X (FEI company, Hillsboro, OR, USA), respectively. The electrical characterization of the device was measured using the HP4155B semiconductor parameter analyzer (HP, Englewood, CO, USA) and a probe station.

## 3. Results and Discussion

**Figure 1:**
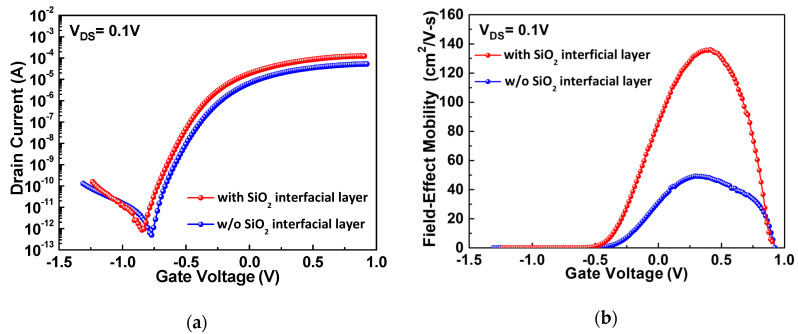
(**a**) *I_DS_-V_GS_* and (**b**) *μ_FE_-V_GS_* characteristics of the top-gate SnO_2_ TFTs with and without an SiO_2_ interfacial layer.

Figure 1a presents the drain-source current versus gate-source voltage (*I_DS_-V_GS_*) characteristics of the top-gate TFTs with and without the SiO_2_ interfacial layer between the SnO_2_ channel and the HfO_2_ gate dielectric. The devices, with and without the ultra-thin SiO_2_, exhibit good I_ON_/I_OFF_s of 1.5 × 10^8^ and 1 × 10^8^, respectively, and sharp turn-on SS values of 108 and 117 mV/dec, respectively. The interface trap density (*D_it_*) can be calculated from SS [29,30]:

(1) $$D_{it}=\frac{1}{q}\left(\frac{SS}{\;kT/q\times \;ln10}-1\right)C_{ox}-\frac{C_{dep}}{q},$$

where *C_dep_* is the depletion capacitance. A *D_it_* of 5.5 × 10^12^ eV^−1^cm^−2^ is obtained, which is higher than the high-κ/silicon transistor. Further interface improvement can increase the *SS* and *μ_FE_*.

Figure 1b depicts the *μ_FE_-V_GS_* characteristics of these devices. The *μ_FE_* was obtained by a standard method used in silicon IC from the trans-conductance (*g_m_*) at a small *V_DS_* of 0.1 V:

(2) $$\mu_{FE}=\frac{g_{m}}{\left(W_{G}/L_{G}\right)C_{ox}V_{DS}},$$

where *W_G_*, *L_G_*, and *C_ox_* are the gate width, gate length, and oxide capacitance, respectively. The *C_ox_* was obtained from the measured *C-V* characteristics divided by the area of the Ni/HfO_2_/SiO_2_/Al MIM device on the same chip. The SnO_2_ TFT with an SiO_2_ interfacial layer has a *μ_FE_* as high as 136 cm^2^/Vs, which is significantly higher than the 49.3 cm^2^/Vs for the device without the SiO_2_ layer. This is the highest *μ_FE_* value for top-gate TFTs made on an amorphous SiO_2_ substrate and processed at a temperature of ≤ 400 °C [21,22,23,24,25].

**Figure 2:**
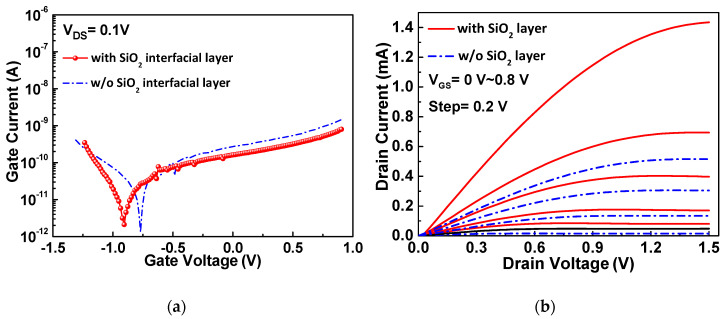
(**a**) *I_GS_-V_GS_* and (**b**) *I_DS_-V_DS_* characteristics of the top-gate SnO_2_ with and without an SiO_2_ interfacial layer.

To understand the significantly better the *I_DS_* and *μ_FE_* data for TFTs with an ultra-thin SiO_2_ layer, we further measured the gate-source current versus gate-source voltage (*I_GS_-V_GS_*) characteristics. As shown in Figure 2a, the gate leakage current does not demonstrate a significant difference between these two devices because the interfacial SiO_2_ layer was only 3 nm thick, and much thinner than the high-κ HfO_2_, which had a thickness of 50 nm. The *I_DS_* versus the drain-source voltage (*I_DS_-V_DS_*) characteristics are presented in Figure 2b. The TFT device with the ultra-thin SiO_2_ layer exhibits a higher I*_DS_* than the TFT without it, which is consistent with the *I_DS_-V_GS_* and *μ_FE_-V_GS_* data presented in Figure 1a,b, because the higher *I_DS_* leads to a higher *μ_FE_* value.

**Figure 3:**
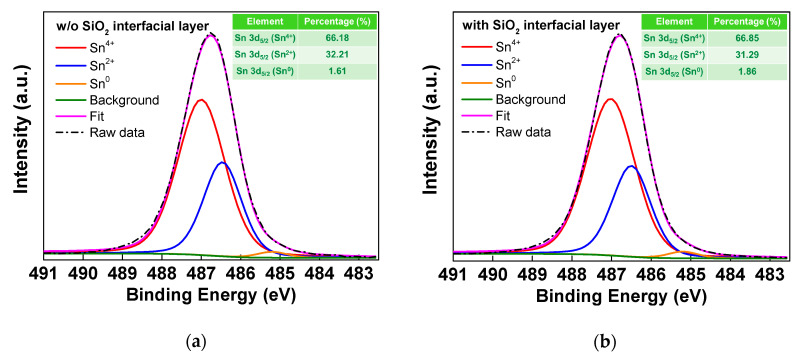
The XPS spectra of Sn 3d_5/2_ in the SnO_2_ layer of the TFT devices (**a**) without and (**b**) with an SiO_2_ interfacial layer.

An XPS analysis was performed on both the HfO_2_/SiO_2_/SnO_2_ and the HfO_2_/SnO_2_ stacks. Before the analysis, both samples were sputter-etched from HfO_2_ to SnO_2_ at a slow rate of 0.1 nm/s. As shown in Figure 3, the Sn 3d_5/2_ spectrum of the SnO_x_ layer is split into three peaks: Sn^4+^, Sn^2+^, and Sn^0^. The binding energies of the Sn^4+^, Sn^2+^, and Sn^0^ peaks were 487, 486.5, and 485.2 eV, respectively. The intensity of Sn^2+^ is related to the p-type SnO TFT [18]. By contrast, Sn^4+^ conducts electrons for n-type TFTs [11,12,13,14,15,16]. As the results obtained using XPS analysis do not indicate obvious differences between these two samples, the inserted SiO_2_ interfacial layer has little effect on the chemical composition of the SnO_x_ channel layer.

**Figure 4:**
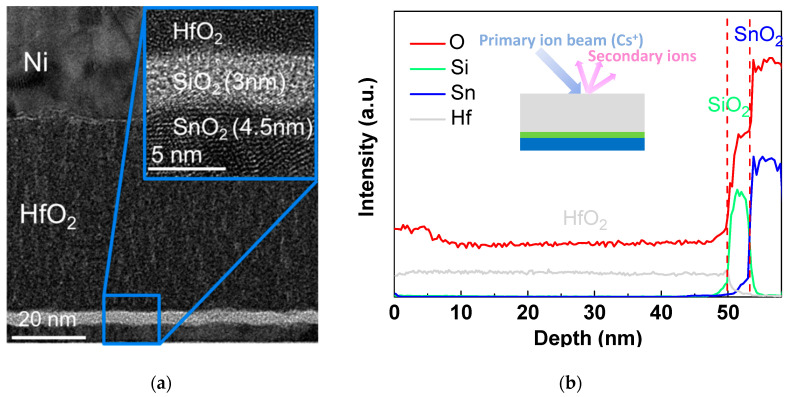
(**a**) The cross-sectional TEM image and (**b**) SIMS depth profiles of the SnO_2_ TFT with an SiO_2_ interfacial layer.

We further investigated the HfO_2_/SiO_2_/SnO_2_ stack through TEM and SIMS measurements. Figure 4a displays the cross-sectional TEM image of the SnO_2_ TFT with an SiO_2_ interfacial layer, where the thicknesses of HfO_2_, SiO_2_, and SnO_2_ were 50, 3, and 4.5 nm, respectively. The distributions of the Sn, Si, Hf, and O atoms in the gate stack and channel layer are depicted from the SIMS depth profiles in Figure 4b. An SiO_2_ interfacial layer was clearly observed in both the TEM and SIMS analyses.

**Figure 5:**
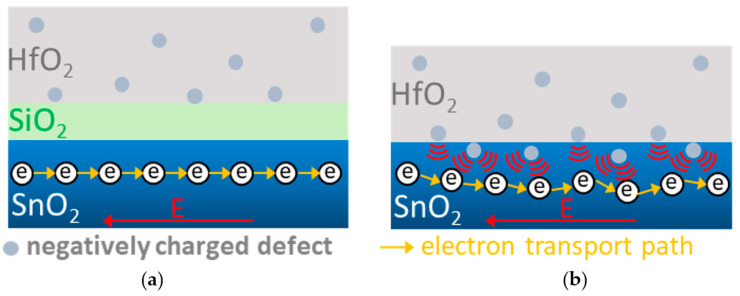
The schematic diagrams for electron transport in (**a**) with (**b**) without an SiO_2_ interfacial layer. Negative charges formed in HfO_2_ for a device without an SiO_2_ layer will increase the electron scattering and lower the mobility.

It is important to note that the extra SiO_2_ interfacial layer will increase the thickness of the gate dielectric slightly and theoretically lead to a slightly higher transistor threshold voltage (V_TH_) than the device without the SiO_2_ layer. However, the *I_DS_-V_GS_* characteristics of the SnO_2_ devices in Figure 1a display a contrary result. Thus, the increased V_TH_ for the device without the interfacial SiO_2_ layer is due to the extra negative charges formed in HfO_2._ These negative charges may also exist in the HfO_2_/SnO_2_ interface because the interface charges are strongly related to *SS* [22], which improves with the extra SiO_2_ interfacial layer, as shown in Figure 1a. Further, such negative charges in the HfO_2_ and HfO_2_/SnO_2_ interfaces can cause electron scattering and degrade the mobility [31,32], as shown in Figure 1b. It is known that the high-κ gate dielectric has defects, especially when formed at low temperatures. The negative charges formed in the HfO_2_ and HfO_2_/SnO_2_ interfaces cause channel electron scattering and mobility degradation, which can be observed in the schematic diagrams illustrated in Figure 5a,b. The device with the SiO_2_ interfacial layer has less negative charge scattering in HfO_2_ and the interface because of the separation of the SiO_2_ layer, which results in a higher mobility and *I_DS_*.

**Figure 6:**
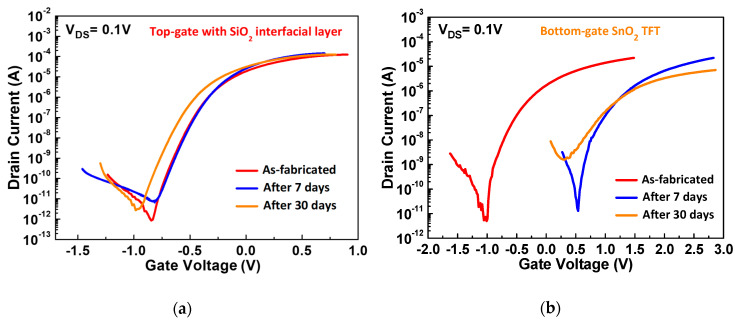
The *I_DS_-V_GS_* characteristics of the (**a**) top-gate and (**b**) bottom-gate SnO_2_ TFT devices measured as-fabricated after 7 days and after 30 days of exposure to ambient air.

The moisture degradation of TFT devices is a significant issue for an IC. Figure 6 illustrates the *I_DS_-V_GS_* characteristics for the as-fabricated top-gate coplanar and bottom-gate staggered SnO_2_ TFTs in ambient air after 7 days and 30 days of exposure to air. The *I_DS_-V_GS_* characteristics of the bottom-gate SnO_2_ TFT are shifted as high as 1.5 V after 7 days of exposure, and the I_OFF_, SS, and I_ON_ further degrade significantly after 30 days of exposure to air. This is because the top SnO_2_ layer can react with H_2_O molecules in the air and form Sn-OH bonds [14,19,20], resulting in charged defects that lower the *I_DS_* and *μ_FE_*. The penetration of OH^-^ into the SnO_2_ could also form defects and lead to a higher I_OFF_ by defect conduction [26]. In sharp contrast, only a slight V_TH_ shift of −0.09 V was observed in the top-gate device because the gate dielectric HfO_2_ layer can behave as a passivation layer on the SnO_2_ channel layer. The slight V**_TH_** shift might be attributed to the intrinsic defects of the HfO_2_ layer and the charge trapping and de-trapping phenomena of those defects [33,34].

In Table 1, we summarize the important device characteristics and compare them with the published data on top-gate TFTs made on amorphous SiO_2_ substrates [21,22,23,24,25]. Our device with an ultra-thin channel thickness of 4.5 nm exhibits the highest *μ_FE_*, a sharp *SS* for low-voltage operation, and a sufficiently large I_ON_/I_OFF_, which are crucial for display, low-leakage DRAM access transistors, and monolithic 3D IC applications. Further improvement of *μ_FE_* and *SS* may be reachable by using a thicker SnO_2_ layer than the 4.5 nm thickness and a Fin Field-Effect Transistor (FinFET) or gate-all-around structure, respectively.

## 4. Conclusions

An excellent device integrity was achieved for a top-gate TFT made on an amorphous SiO_2_ substrate using a low process temperature of 350 °C with a high *μ_FE_* of 136 cm^2^/Vs, a sharp SS of 108 mV/dec for low-voltage operations, and a sufficiently large I_ON_/I_OFF_ of 1.5 × 10^8^. Such a top-gate structure is preferred for monolithic IC as compared to bottom-gate devices. In addition, a much better resistance to moisture can be achieved than in the bottom-gate device without passivation. Such a superb device performance is strongly related to the inserted ultra-thin SiO_2_ layer between the HfO_2_ and SnO_2_. The outstanding device performance with top-gate structure is a crucial technology for future-generation high-resolution displays, low-leakage DRAM access transistors, and monolithic 3D brain-mimicking ICs.

## Figures and Tables

**Table 1:** Important device performance comparison of various top-gate TFT devices on SiO_2_ substrate.

Channel Materials	Channel Thickness (nm)	*μ_FE_* (cm^2^/V·s) @V*_DS_*(V)	I_ON_/I_OFF_	SS (mV/Decade)
a-Si [21]	100	0.9 @ 0.1	10^5^	380
Poly-Si [22]	100	40 @ 0.1	1.5 × 10^6^	310
IGZO [23]	40	11.44 @ 10	10^8^	360
ZnO [24]	50	16.8 @ 0.1	2.4 × 10^9^	102
SnO_2_ [25]	30	4.43 @ 1	4.19 × 10^6^	300
SnO_2_ this work	4.5	136 @ 0.1	1.5 × 10^8^	108

## References

[1] Klasens H.A., Koelmans H. (1964). A tin oxide field-effect transistor. Solid State Electron..

[2] Kwon J.Y., Son K.S., Jung J.S., Kim T.S., Ryu A.K., Park K.B., Yoo B.W., Kim J.W., Lee Y.G., Park K.C. (2008). Bottom-gate Gallium Indium Zinc Oxide thin-film transistor array for high-resolution AMOLED display. IEEE Electron. Device Lett..

[3] Xu H., Luo D., Li M., Xu M., Zou J., Tao H., Lan L., Wang L., Peng J., Cao Y. (2014). A flexible AMOLED display on the PEN substrate driven by oxide thin-film transistors using anodized aluminium oxide as dielectric. J. Mater. Chem. C.

[4] Fortunato E., Barquinha P., Martins R. (2012). Oxide semiconductor thin-film transistors: A review of recent advances. Adv. Mater..

[5] Nomura K., Ohta H., Takagi A., Kamiya T., Hirano M., Hosono H. (2004). Room-temperature fabrication of transparent flexible thin-film transistors using amorphous oxide semiconductors. Nature.

[6] Sekitani T., Zschieschang U., Klauk H., Someya T. (2010). Flexible organic transistors and circuits with extreme bending stability. Nat. Mater..

[7] Su N.C., Wang S.J., Huang C.C., Chen Y.H., Huang H.Y., Chiang C.K., Chin A. (2010). Low-voltage-driven flexible InGaZnO thin-film transistor with small subthreshold swing. IEEE Electron. Device Lett..

[8] Zan H.W., Li C.H., Yeh C.C., Dai M.Z., Meng H.F., Tsai C.C. (2011). Room-temperature-operated sensitive hybrid gas sensor based on amorphous indium gallium zinc oxide thin-film transistors. Appl. Phys. Lett..

[9] Kim K.S., Ahn C.H., Jung S.H., Cho S.W., Cho H.K. (2018). Toward adequate operation of amorphous oxide thin-film transistors for low-concentration gas detection. ACS Appl. Mater. Interfaces.

[10] Liao L., Zhang Z., Yan B., Zheng Z., Bao Q.L., Wu T., Li C.M., Shen Z.X., Zhang J.X., Gong H. (2009). Multifunctional CuO nanowire devices: P-type field effect transistors and CO gas sensors. Nanotechnology.

[11] Shih C.W., Chin A., Lu C.F., Yi S.H. (2015). Extremely high mobility ultra-thin metal-oxide with ns^2^np^2^ configuration. Int. Electron. Devices Meeting (IEDM) Technol. Dig..

[12] Chin A., Yen T.J., Shih C.W., Chen Y.D. (2019). High mobility metal-oxide devices for display SoP and 3D brain-mimicking IC. Proc. Int. Disp. Workshops.

[13] Chin A., Chen Y.D. Technologies toward three-dimensional brain-mimicking IC architecture. Proceedings of the 2019 Electron Devices Technology and Manufacturing Conference (EDTM).

[14] Shih C.W., Chin A. (2017). Remarkably High mobility thin-film transistor on flexible substrate by novel passivation material. Sci. Rep..

[15] Shih C.W., Chin A., Lu C.F., Su W.F. (2016). Remarkably high mobility ultra-thin-film metal-oxide transistor with strongly overlapped orbitals. Sci. Rep..

[16] Shih C.W., Yen T.J., Chin A., Lu C.F., Su W.F. (2019). Low-temperature processed tin oxide transistor with ultraviolet irradiation. IEEE Electron Device Lett..

[17] Shih C.W., Chin A. (2016). New material transistor with record-high field-effect mobility among wide-band-gap semiconductors. ACS Appl. Mater. Interfaces.

[18] Shih C.W., Chin A., Lu C.F., Su W.F. (2018). Remarkably high hole mobility metal-oxide thin-film transistors. Sci. Rep..

[19] Yoo D.Y., Chong E., Kim D.H., Ju B.K., Lee S.Y. (2012). Effect of magnesium oxide passivation on the performance of amorphous indium–gallium–zinc-oxide thin film transistors. Thin Solid Film.

[20] Chowdhury M.D.H., Mativenga M., Um J.G., Mruthyunjaya R.K., Heiler G.N., Tredwell T.J., Jang J. (2015). Effect of SiO_2_ and SiO_2_/SiN_x_ passivation on the stability of amorphous indium–gallium zinc-oxide thin-film transistors under high humidity. IEEE Trans. Electron Devices.

[21] Han L., Huang Y., Sturm J.C., Wagner S. (2011). Self-aligned top-gate coplanar a-Si:H thin-film transistors with a SiO_2_–Silicone hybrid gate dielectric. IEEE Electron Device Lett..

[22] Hung B.F., Chiang K.C., Huang C.C., Chin A., McAlister S.P. (2005). High-performance poly-silicon TFTs incorporating LaAlO_3_ as the gate dielectric. IEEE Electron Device Lett..

[23] Zhang S., Shao Y. Technology issues for self-aligned top-gate amorphous metal oxide thin-film transistors. Proceedings of the IEEE International Conference on Electron Devices and Solid State Circuits.

[24] Deng S. (2019). Gate Insulator engineering in top-gated indium-tin-oxide-stabilized ZnO thin-film transistors. IEEE Electron. Device Lett..

[25] Lim D., Jeon Y., Kim M., Kim Y., Kim S. (2016). Electrical characteristics of SnO_2_ thin-film transistors fabricated on bendable substrates using reactive magnetron sputtering. J. Nanosci. Nanotechnol..

[26] Saha J.K., Bukke R.N., Mude N.N., Jang J. (2020). Remarkable Stability Improvement of ZnO TFT with Al_2_O_3_ Gate Insulator by Yttrium Passivation with Spray Pyrolysis. Nanomaterials.

[27] Zhu S., Chen J., Li M.-F., Lee S.J., Singh J., Zhu C.X., Du A., Tung C.H., Chin A., Kwong D.L. (2004). N-type Schottky barrier source/drain MOSFET using ytterbium silicide. IEEE Electron Device Lett..

[28] Zhu S., Yu H.Y., Whang S.J., Chen J.H., Shen C., Zhu C., Lee S.J., Li M.F., Chan D.S.H., Yoo W.J. (2004). Schottky-barrier S/D MOSFETs with high-k gate dielectrics and metal gate electrode. IEEE Electron Device Lett..

[29] Chang M.F., Lee P.T., McAlister S.P., Chin A. (2008). Low sub-threshold swing HfLaO/Pentacene organic thin film transistors. IEEE Electron Device Lett..

[30] Kim M.S., Kim H.T., Chi S.S., Kim T.E., Shin H.T., Kang K.W., Park H.S., Kim D.J., Min K.S., Kang D.W. (2003). Distribution of interface states in MOS systems extracted by the subthreshold current in MOSFETs under optical illumination. J. Korean Phys. Soc..

[31] Fischetti M.V., Neumayer D.A., Cartier E.A. (2001). Effective electron mobility in Si inversion layers in metal-oxide-semiconductor systems with a high-kappa insulator: The role of remote phonon scattering. J. Appl. Phys..

[32] Maitra K. (2007). Impact of metal gates on remote phonon scattering in titanium nitride/hafnium dioxide n-channel metal–oxide–semiconductor field effect transistors–low temperature electron mobility study. J. Appl. Phys..

[33] Zafar S., Callegari A., Gusev E., Fischetti M.V. (2003). Charge trapping related threshold voltage instabilities in high permittivity gate dielectric stacks. J. Appl. Phys..

[34] Lee B.H. Intrinsic characteristics of high-k devices and implications of fast transient charging effects (FTCE). Proceedings of the IEDM Technical Digest. IEEE International Electron Devices Meeting.

